# The effects of communicating illness diagnostic and treatment information and C‐reactive protein test results on people's antibiotic expectations

**DOI:** 10.1111/bjhp.70020

**Published:** 2025-09-02

**Authors:** Andriana Theodoropoulou, Matteo Lisi, Jonathan Rolision, Miroslav Sirota

**Affiliations:** ^1^ Department of Psychology University of Essex Colchester UK; ^2^ Essex ESNEFT Psychological Research Unit for Behaviour, Health and Wellbeing University of Essex Colchester UK; ^3^ Department of Psychology Royal Holloway London UK

**Keywords:** antibiotic expectations, communication strategies, diagnostic uncertainty, point‐of‐care tests, respiratory tract infections, signal detection theory

## Abstract

**Objectives:**

Patients' expectations for antibiotics are among the strongest predictors of clinicians' decisions to overprescribe antibiotics. In this registered report, we used a signal detection theory framework to investigate the experimental effects of the communication interventions that family physicians can use to reduce patients' diagnostic uncertainty, and consequently, their antibiotic expectations.

**Methods:**

UK participants (*N* = 769) read hypothetical consultations for respiratory tract infections and were randomly assigned to one of three conditions: standard information (control), recommended information about the nature of the illness and antibiotic efficacy (recommended communication) or recommended information accompanied by point‐of‐care test results (recommended communication and CRP). Using a multilevel Bayesian probit regression, we estimated both decision bias (criterion) and sensitivity (d‐prime).

**Results:**

Aligned with our bias hypotheses, participants displayed a more liberal antibiotic bias in the control condition compared to both the recommended communication (Δ*c* = −1.34, 95% CI [−1.57, −1.11]) and the recommended communication and CRP (Δ*c* = −1.73, 95% CI [−1.99, −1.48]) conditions. They also showed greater liberal bias in the recommended communication condition compared to the recommended communication and CRP condition (Δ*c* = −0.39, 95% CI [−0.65, 0.13]). Aligned with our sensitivity hypotheses, participants displayed significantly higher sensitivity in both the recommended communication (Δ*d*' = 2.34, 95% CI [1.92, 2.79]) and the recommended communication and CRP (Δ*d*' = 2.49, 95% CI [2.08, 2.95]) conditions compared to control.

**Conclusions:**

Simple, evidence‐based communication strategies—particularly when combined with diagnostic test results—can reduce antibiotic expectations, offering practical tools for clinicians to support appropriate prescribing.

## INTRODUCTION

Antimicrobial resistance is currently one of the biggest global health threats (Centers for Disease Control and Prevention, 2019; World Health Organization, 2014) and is estimated to result in 10 million deaths per annum by 2050 if no action is taken (O'Neill, 2014, 2016). Around 80% of antibiotics are inappropriately prescribed in primary care to patients consulting for symptoms of respiratory tract infections (Davies, 2018; Goossens et al., 2005; Gulliford et al., 2014; Shapiro et al., 2014). Patients' antibiotic expectations and requests have consistently been found among the strongest predictors of clinicians' decisions to prescribe antibiotics (McNulty et al., 2013; Sirota et al., 2017; Welschen et al., 2004). To decrease over‐prescribing, we propose here to experimentally investigate two pieces of information that doctors can communicate to their patients to reduce patients' diagnostic uncertainty, and consequently, their expectations for antibiotics: clinicians' judgement about the nature of the illness and the result of a point‐of‐care test.

Patients' diagnostic uncertainty about the nature of the illness and the efficacy of antibiotics has gathered strong empirical and theoretical support as the main driver of antibiotic expectations. In many cases, patients expect antibiotics for conditions that do not require them, such as viral infections (Braun & Fowles, 2000; McNulty et al., 2013, 2019; Welschen et al., 2004). Diagnostic uncertainty can also concern the clinical symptoms and whether they manifest a viral or bacterial infection. For example, uncertainty regarding the nature of their illness predicted people's expectations for antibiotics for their recently experienced symptoms of a cold (Thorpe et al., 2021). Decreasing patients' diagnostic uncertainty is therefore essential to lowering their antibiotic expectations.

Communicating clinical judgements about the nature of the illness, as well as information about the efficacy of antibiotics, could be an effective communication intervention for decreasing patients' diagnostic uncertainty and their antibiotic expectations. This is also in line with current NICE clinical guidelines about the management of respiratory tract infections that recommend that both information regarding the clinical judgement about the illness and the efficacy of antibiotics should be communicated to patients (NICE, 2021). The majority of studies focusing on patient communication strategies, such as public health campaigns, enhanced patient communication and patient leaflets, have primarily focused on prescribers reducing antibiotic prescribing in primary care (Arnold & Straus, 2005; Köchling et al., 2018; Little et al., 2019; Macfarlane et al., 2002; Tonkin‐Crine et al., 2017). Recent evidence, however, has also emerged for the role of communicating clinical information on reducing patients' antibiotic expectations (Sirota et al., 2022; Thorpe et al., 2020a, 2020b, 2021). For instance, decreasing diagnostic uncertainty by clarifying when antibiotics are needed significantly reduced people's expectations and requests for antibiotics in a UK sample (Sirota et al., 2022). Similarly, recent robust experimental evidence showed that providing a clinician's judgement about the illness aetiology of the symptoms, and in some cases including a diagnostic test result, significantly decreased people's antibiotic expectations in hypothetical consultations (Thorpe et al., 2020a, 2020b, 2021).

Communicating the results of a point‐of‐care test, such as C‐reactive protein (CRP), could also be an effective strategy for decreasing the diagnostic uncertainty of patients. Even though point‐of‐care tests are not routinely used in the United Kingdom (John et al., 2022), they are increasingly popular in clinical practices because the evidence supports their role in reducing antibiotic prescribing in both primary care and hospital settings (Andreeva & Melbye, 2014; Cals et al., 2009, 2010; Köchling et al., 2018; Little et al., 2013; Tonkin‐Crine et al., 2017). CRP, in particular, has been a robust measure for improving the assessment of respiratory tract infections and increasing diagnostic certainty (Andreeva & Melbye, 2014; Cals et al., 2009, 2010). Although limited evidence exists regarding the UK general public's views of such tests, the evidence so far is favourable with respect to a perceived improvement in the quality of health care that these tests would bring to the hospital and home environment (Kaman et al., 2013; Tonkin‐Crine et al., 2014). However, little is known about the role of such tests in reducing people's antibiotic expectations, and only recently has experimental evidence suggested that including a diagnostic test result significantly decreases people's antibiotic expectations in the United Kingdom (Thorpe et al., 2020a).

### Present study

Here, we aimed to experimentally investigate the communication interventions that family physicians can use to reduce patients' diagnostic uncertainty, and consequently, their antibiotic expectations in respiratory tract infections while using a signal detection theory framework (Lynn & Barrett, 2014; Sirota et al., 2022). Specifically, we presented people with hypothetical consultations with a family physician for hypothetical respiratory tract infections. Participants received either standard information (control group), or recommended information about the nature of the illness and antibiotic inefficacy (recommended communication) or recommended information accompanied by the results of the point‐of‐care test (recommended communication and CRP). To approximate the higher real‐world rates of encountering viral versus bacterial illnesses for symptoms of respiratory tract infections, we modelled the decision environments so that only 25% of the scenarios presented in all conditions required antibiotic treatment (Creer et al., 2006).

We hypothesised that participants would display a more liberal antibiotic bias (i.e., resulting in increased antibiotic expectations relative to the criterion location) in the control condition compared to the recommended communication condition (Hypothesis 1). We also expected that participants would display a more liberal antibiotic bias in the recommended communication condition compared to the recommended communication and CRP condition (Hypothesis 2). Similarly, we expected that participants would display a more liberal bias in the control condition compared to the recommended communication and CRP condition (Hypothesis 3). Finally, we expected that participants would display lower sensitivity (i.e., reduced ability to discriminate correctly between the different scenarios whether antibiotics are needed or not) in the control condition compared to either the recommended communication condition or the recommended communication and CRP condition (Hypothesis 4).

## METHODS

### Ethics

The proposed research complied with all relevant ethical regulations. It received ethical approval from the Ethics Committee at the University of Essex (ref: ETH2324‐0194). Informed consent was obtained from all participants prior to starting the study online. Participants were also remunerated £0.70 for their time (based on an estimated study completion time of 5 min at a Prolific rate of £8.40/h).

### Design

The random allocation of the participants was done by the Qualtrics built‐in randomizer with the ‘Evenly Present Elements’ feature, which balances conditions presentation based on a least‐fill randomization algorithm to ensure that all conditions are displayed evenly across participants while maintaining randomness in selection. Thus, this study was a single‐blind randomized controlled trial, with participants blind to the intervention conditions. Participants were randomly allocated to one of the three conditions: control condition (no additional communication provided), recommended communication condition (additional communication provided regarding the nature of the illness and efficacy of antibiotics) and recommended communication and CRP condition (additional communication provided regarding the nature of the illness and the efficacy of antibiotics, as well as the results of a CRP test) with an allocated ratio of 1:1:1. Expectations for antibiotics were the dependent variable. Participants read 16 within‐subjects hypothetical medical scenarios (the base rate was set such that antibiotics were needed in 25% of the scenarios presented) and were asked to report their need for antibiotics as a treatment via a binary (yes or no) response for each of them. From the responses to the antibiotic expectations variable, we calculated the signal detection model parameters: bias and sensitivity.

### Procedure

After providing informed consent and randomization, participants expressed their antibiotic expectations for each of the 16 hypothetical medical vignettes (randomly presented with a fixed attention check question). The vignettes described a consultation with a physician for symptoms of respiratory tract infections modelled after the NHS and NICE clinical guidelines for situations where antibiotics are clinically justified or not. Due to the COVID‐19 pandemic, all participants were told at the beginning of the task that COVID‐19 could not account for their symptoms, and they were asked to indicate ‘yes’ in a following question showing that they had read and understood the above information.

The scenarios in which antibiotics were not needed (75% of the scenarios presented in all three conditions) were modelled after cases of acute bronchitis where antibiotics should not be prescribed. Acute bronchitis is a self‐limited respiratory tract infection with a cough as the primary symptom (Albert, 2010). Approximately 90% of acute bronchitis infections are caused by viruses (Gonzales et al., 2001; Worrall, 2008) and the NICE clinical guidelines recommend that antibiotics should not be prescribed to patients consulting for symptoms associated with bronchitis (NICE, 2019a). Conversely, the scenarios in which antibiotics were needed (25% of the scenarios presented in all three conditions) were modelled after clinical cases of community‐acquired pneumonia where antibiotics should be prescribed. Pneumonia is a lower respiratory tract infection that is most commonly caused (in around 90% of cases) by a bacterial infection (Jain et al., 2015; Lim et al., 2009). However, due to its high mortality rate, the clinical guidelines state that all people exhibiting symptoms of community‐acquired pneumonia should be prescribed antibiotics (NICE, 2019b).

In all three conditions, all participants received a description of the symptoms and illness duration and a description of the physical chest examination. All the scenarios had been pre‐tested in prior studies to ensure that they were qualitatively similar. Specifically, all scenarios had the following set of symptoms that stayed constant: sore throat, runny nose, muscle aches and general fatigue. Moreover, the following pieces of clinical information were present in all scenarios, but were varied: *illness duration, cough, phlegm, temperature, breathlessness and physical chest examination* (see Table 1 for an example scenario modelled after a viral illness). In the control condition, participants were presented in each scenario with only the clinical information regarding the symptoms, illness duration and chest examination (see Table 1). In the recommended communication condition, participants were also presented with additional clinical information regarding the nature of the illness and the efficacy of antibiotics by their GP (i.e., ‘Given your symptoms, they think that a viral respiratory tract infection is the cause. They explain that antibiotics are only effective for bacterial infections and that they are not an effective treatment in this case’). In the recommended communication and CRP condition, participants were presented with both the additional clinical information regarding the nature of the illnesses and the efficacy of antibiotics, as well as the results of a CRP test by their GP (i.e., ‘They explain the results of your rapid blood test. They tell you that your results show that there is no inflammation in your body. Given your symptoms and the results of the test, they think that a viral respiratory tract infection is the cause. They explain that antibiotics are only effective for bacterial infections and that they are not an effective treatment in this case’). After each scenario, participants were asked to report their perceived need for antibiotics as a treatment (i.e., ‘I need antibiotics’) via a binary (yes or no) response scale.

**TABLE 1:** An example antibiotic scenario of a viral illness.

For the past **five days** you have been feeling ill. You have had a **sore throat** and a **runny nose**, and you have been experiencing **muscle aches** and **general fatigue**. You have also developed a **dry hacking cough**. However, you have a **normal temperature** and **no breathlessness**. Upon examination, your GP tells you that your lungs sound **clear**

Lastly, participants were asked to complete some socio‐demographic questions (i.e., age, sex, education, occupation and language) at the end of the study following the intervention completion. Participants were then debriefed. The whole study took around 5 min to complete.

### Analysis plan

We ran a multilevel Bayesian generalized linear model (GLM), with a probit link function to estimate the group‐level signal detection model parameters: bias (participants' propensity to expect or not antibiotics in different clinical cases; it is the distance of the decision criterion from the noise distribution, also referred to as *c*) and sensitivity (participants' ability to discriminate between different clinical cases of whether antibiotics are needed or not; it is the distance between signal and noise distribution, expressed in standard deviations, also referred to as *d*') from their responses to the repeated binary outcome measure of antibiotic expectations (see Power Analysis script for details).

The signal detection theory parameters have a one‐to‐one mapping with the parameters of the generalized linear model (Knoblauch & Maloney, 2012). In the GLM formulation, we included a dummy predictor (1 for signal present, 0 for absent), where the slope of this predictor corresponds to *d'*, the sensitivity measure in equal‐variance Gaussian SDT. Similarly, the intercept parameter in the probit GLM corresponds to the negative of the SDT decision criterion (*c* = −*β*_0_). These mappings allowed us to derive the signal detection parameters directly from the probit model, enabling the estimation of bias and sensitivity while assuming equal variance for signal and noise distributions. By analysing the posterior distribution of the multilevel Bayesian probit model signal detection parameters, bias and sensitivity, we obtained credible intervals around these transformed quantities across conditions, which served as the basis for our inferential decisions.

Specifically, we estimated the signal detection theory parameters for the three communication conditions (control, recommended communication, recommended communication and CRP) to test whether participants' propensity to expect antibiotics (i.e., the location of the decision criterion), as well as participants' ability to discriminate correctly between the different scenarios (i.e., the sensitivity) change as a function of the information provided. We estimated the 95% Bayesian credible interval of the difference in the criterion and the sensitivity between the three communication conditions (more specifically, we used highest posterior density interval, HPDI). We considered a difference significant when its 95% credible interval did not include zero.

We additionally conducted model diagnostic and fit checks to assess model fit and confirm convergence. Specifically, we calculated the R hat statistic (Gelman & Rubin, 1992) to confirm that there were no divergent transitions, and we conducted posterior predictive checks to compare the observed with the simulated data to assess how well the model reproduces the observed patterns.

### Sampling plan

Participants were recruited online via the participant research platform Prolific. Eligible panel members needed to meet the following criteria to take part in our study: (i) be UK residents, (ii) have a Prolific approval rate of more than 90% and (iii) not have taken part in any of our previous studies. A balanced sample in terms of sex was also selected in the Prolific screening criteria.

**FIGURE 1:**
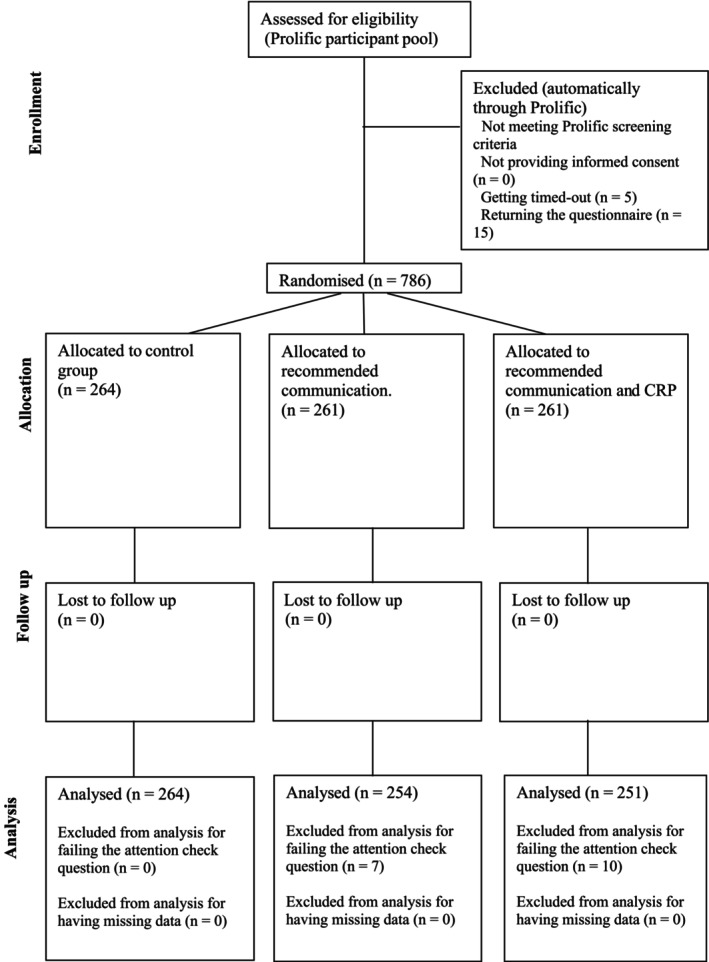
CONSORT diagram of participant flow.

We aimed to recruit a minimum analytical sample size of *N* = 750 (250 valid responses per condition). We initially recruited 759 responses, and when the desired analytical sample size had not been achieved, the primary researcher continued data collection through Prolific in increments of nine responses until 250 valid responses were reached per condition. Given the online data collection, we followed a per‐protocol approach; participants with incomplete data and participants who failed the attention check question were excluded from the final analytical sample size to avoid random responding and increase the reliability of our findings (see Figure 1 for participant flow). Furthermore, to maintain objectivity during the data collection phase, data were only analysed after all conditions were fully recruited, with no interim analysis planned.

The sample size was based on a priori power analyses to test all our hypotheses. In order to estimate the sample size required to test our hypotheses, we used a simulation approach. The effect sizes and parameters of the simulations (fixed‐effects coefficients in the control condition and variance–covariance matrix for random effects) were set to values for the bias and sensitivity estimated in our previous studies that used the same method (The detailed Power Analysis script can be found here: https://osf.io/fd2y4/?view_only=f5b887fc5d5e4272b465f5c1b1a4e883).

In multilevel models with binary outcomes, such as the one employed in our study, standard effect sizes are not uniquely defined. We adapted Cohen's *d* to our context by considering effect sizes as the mean difference between conditions divided by the standard deviation of the random effects (which represent subject‐specific deviations from the mean difference). This adaptation is akin to Cohen's *d* but is more conservative. The reason for this conservatism lies in the inherent uncertainty in estimating random effects (i.e., parameters of individual subjects) from a finite dataset. Therefore, the actual denominator of our effect size was larger than what was directly set in the simulation, leading to a ‘true’ Cohen's *d* that was smaller than the nominal effect size we specified. This subtlety ensured a conservative estimate in our simulations.

We chose *d* = 0.2 as the smallest effect size of interest for Hypotheses 1, 2 and 4, which corresponded to sensitivity (*d*‐prime) and bias (criterion) effects. The effect of information provision on antibiotic expectations measured in a single vignette has been shown to be larger than *d* = 0.2. For instance, prior research (Thorpe et al., 2020b) reported medium to large effects of information provision on people's antibiotic expectations corresponding to Cohen's *d* = 0.52 (Exp. 1) and *d* = 0.85 (Exp. 2). Given our context and our multilevel modelling approach, our chosen effect size was thus more conservative. This threshold allowed us to focus on detecting differences that are both statistically robust and practically meaningful, reflecting our judgement that effects smaller than *d* = 0.2 are unlikely to have practical relevance or meaningful implications for policy decisions regarding antibiotic expectations. For Hypothesis 3, which tested the additive effect of combining communication strategies, we estimated a larger additive effect size (*d* = 0.4). This choice reflects the hypothesis that providing both recommended communication and CRP information would yield a cumulative impact on participants' expectations.

The decision rule in our analysis was to conclude the presence of an effect when the 95% credible intervals did not include zero. In practice, since we used only weakly informative priors (specifically Gaussians with a mean of zero and a standard deviation of one, which roughly constrained the magnitudes of the estimated effects to a plausible range but did not carry any information about their direction), our simulations indicated that this approach effectively corresponded to a false positive rate of 5%. More specifically, for our Bayesian multilevel probit regression model, we specified priors as follows:

The fixed‐effects part of the model is given by:

$$\text{probit}\;\left(Y=1\right)=\beta_{0}+\beta_{1}\;X+\beta_{2}\;\text{condition}+\beta_{3}\;X\times \text{condition}$$

where *X* is a binary indicator (0/1) for indicating whether antibiotics are needed or not, *Y* is a binary yes/no response and condition is a categorical factor with two levels representing our conditions (recommended communication and recommended communication with CRP).

- The prior for *β*_1_, which corresponds to the *d'* (sensitivity) in the baseline condition, was set to *β*_1_ ~ N(2,2), reflecting prior findings with this protocol.
- The prior for *β*_0_, which determines the SDT criterion (*c* = −*β*_0_), was set to *β*_0_ ~ N(−1,1), corresponding to a Gaussian centred on what would be the optimal criterion (*c* = 1) when *d*'= 2 and equal base rates.
- All other coefficients were given weakly regularizing priors: β* ~ N(0,1), constraining estimates to a plausible range without assuming directional effects.

The model was estimated within a multilevel (mixed‐effects) framework, with random intercepts and random slopes for X grouped by participant ID. The variance–covariance matrix for the random effects was assigned a decomposition (decov) prior with a regularization parameter of 2, a concentration parameter of 2 and a scale of 1. This prior imposes moderate regularization, stabilizing the estimation of the covariance structure while allowing sufficient flexibility to capture individual differences. Overall, these priors balance regularization and informativeness, aligning with theoretical expectations of the signal detection framework. A sensitivity analysis was also conducted using alternative priors (e.g., wider Gaussian priors) to confirm the robustness of the results.

Specifically, based on power simulations for detecting an effect size of Cohen's *d* = 0.2 for the differences in the criterion between the control and the recommended communication condition assuming *α* = .05, a sample size of *N* = 250 gave an estimated statistical power of around 90% (Hypothesis 1).

Based on power simulations for detecting an effect size of Cohen's *d* = 0.2 for the differences in the criterion between the recommended communication and the recommended communication and CRP condition assuming *α* = .05, a sample size of *N* = 250 gave an estimated statistical power of around 80% (Hypothesis 2).

Based on power simulations for detecting an effect size of Cohen's *d* = 0.4 for the differences in the criterion between the control and the recommended communication and CRP condition assuming *α* = .05, a sample size of *N* = 250 gave an estimated statistical power of around 100% (Hypothesis 3).

Based on power simulations for detecting an effect size of Cohen's *d* = 0.2 for the differences in the sensitivity between the control condition and the recommended communication or the recommended communication and CRP condition assuming *α* = .05, a sample size of *N* = 250 gave an estimated statistical power of more than 90% (Hypothesis 4).

## RESULTS

### Participants

Based on our registered protocol, we aimed to recruit a minimum of 250 valid responses per condition. This was achieved. In our final analytical sample size of *N* = 769 (control condition *N* = 264; recommended communication condition *N* = 254; recommended communication and CRP condition *N* = 251), 380 participants identified as male, 383 as female and 6 as other. The sample age ranged from 18 to 87 years old (*M* = 45.0, *SD* = 14.3 years). Most participants (94.5%) were native English speakers. Participants' occupation varied as follows: management, professional and related (30.7%), service (6.4%), sales and office (10.3%), construction, extraction and maintenance (1.6%), production, transportation and material moving (2.9%), government (6.4%), retired (12.7%), unemployed (7.7%), student (3.9%) and other (16.9%). Their level of education varied as follows: less than high school (1.2%), high school degree (14.7%), some college (22.5%), undergraduate degree (39.5%), master's degree (17.3%) and doctoral or professional degree (4.8%). The full participant descriptives for each condition are shown in Table 2.

**TABLE 2:** Participant characteristics across conditions.

	Control (*N* = 264)	Communication (*N* = 254)	Communication + CRP (*N* = 251)
Sex			
Female	122 (46.2%)	133 (52.4%)	125 (49.8%)
Male	141 (53.4%)	119 (46.9%)	123 (49.0%)
Other	1 (0.4%)	2 (0.8%)	3 (1.2%)
Age in years, M (SD)	44.7 (14.1)	45.9 (14.4)	44.3 (14.4)
Language			
Native English	245 (92.8%)	239 (94.1%)	243 (96.8%)
Non‐native English	19 (7.2%)	15 (5.9%)	8 (3.2%)
Job			
Management, professional and related	74 (28.0%)	75 (29.5%)	87 (34.7%)
Service	20 (7.6%)	18 (7.1%)	11 (4.4%)
Sales and office	36 (13.6%)	24 (9.5%)	19 (7.6%)
Farming, fishing and forestry	3 (1.1%)	1 (0.4%)	1 (0.4%)
Construction, extraction and maintenance	NA	6 (2.4%)	6 (2.4%)
Production, transportation and material moving	6 (2.3%)	5 (2.0%)	11 (4.4%)
Government	20 (7.6%)	8 (3.2%)	21 (8.4%)
Retired	32 (12.1%)	38 (15.0%)	28 (11.2%)
Unemployed	24 (9.1%)	18 (7.1%)	17 (6.8%)
Student	9 (3.4%)	9 (3.5%)	12 (4.8%)
Other	40 (15.2%)	52 (20.5%)	38 (15.1%)
Education			
Less than High School	6 (2.3%)	3 (1.2%)	NA
High School	32 (12.1%)	39 (15.4%)	42 (16.7%)
Some College	61 (23.1%)	59 (23.2%)	53 (21.1%)
Undergraduate Degree	106 (40.2%)	93 (36.6%)	105 (41.8%)
Master's Degree	43 (16.3%)	53 (20.9%)	37 (14.7%)
Doctoral (PhD) or Professional (JD, MD) Degree	16 (6.1%)	7 (2.8%)	14 (5.6%)

### Model overview

**FIGURE 2:**
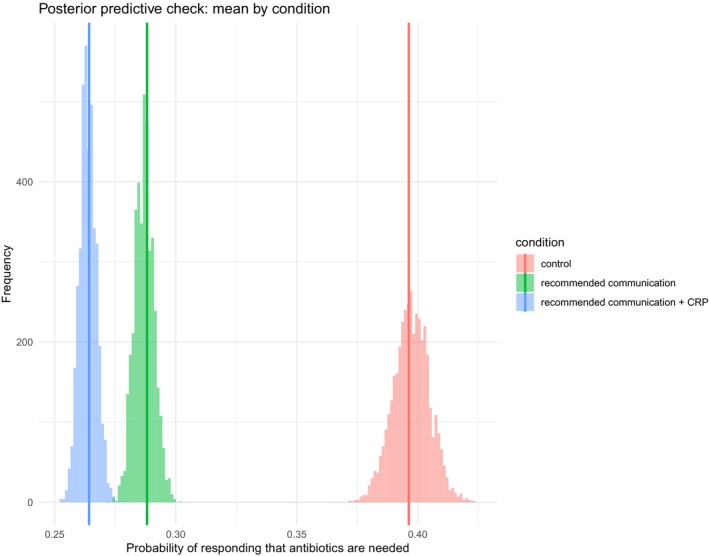
Posterior predictive checks across conditions. The figure shows the mean posterior probability of responding that antibiotics are needed across the three conditions. The vertical bars show the observed means, while the histograms show the posterior predictive samples.

We conducted a pre‐registered multilevel Bayesian generalized linear model (with a probit link function, thus equivalent to a signal detection theory model with a Gaussian distributed latent decision variable) to estimate the group‐level signal detection model parameters: bias (the distance of the decision criterion from the noise distribution) and sensitivity (the distance between signal and noise distribution) from participants' responses to the repeated binary outcome measure of antibiotic expectations. Specifically, we estimated the signal detection theory parameters for the three communication conditions (control, recommended communication, recommended communication and CRP) to test whether participants' propensity to expect or not antibiotics (i.e., the location of the decision criterion), as well as participants' ability to discriminate correctly between the different scenarios (i.e., their sensitivity) change as a function of the information provided. The model converged successfully (R‐hat <1.05 for all parameters) and posterior predictive checks indicated good fit to the data (see Figure 2). To assess the robustness of the results, we also conducted a sensitivity analysis using alternative wider Gaussian priors and priors from a t‐distribution (see Table S1). The key findings remained stable, indicating that results are not driven by prior choices. We then analysed the posterior distribution of the multilevel Bayesian probit model and estimated the 95% Bayesian credible intervals of the difference in the criterion and the sensitivity between the three communication conditions.

### Antibiotic bias

**FIGURE 3:**
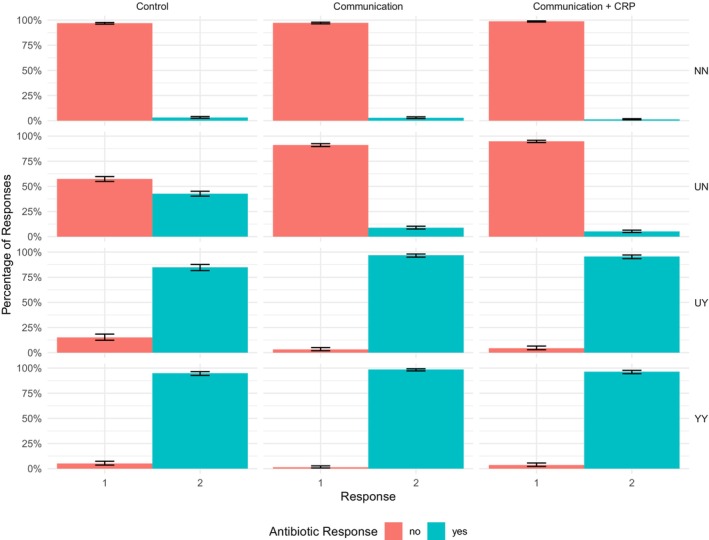
Distribution of antibiotic responses across scenarios and conditions. The figure shows the distribution of antibiotic responses (1 = ‘no’, 2 = ‘yes’) in the four scenario types: certain antibiotics are not needed (NN), uncertain antibiotics are not needed (UN), uncertain antibiotics are needed (UY) and certain antibiotics are needed (YY) in the control, recommended communication and recommended communication and CRP conditions. The black error bars represent the standard error of the mean.

**FIGURE 4:**
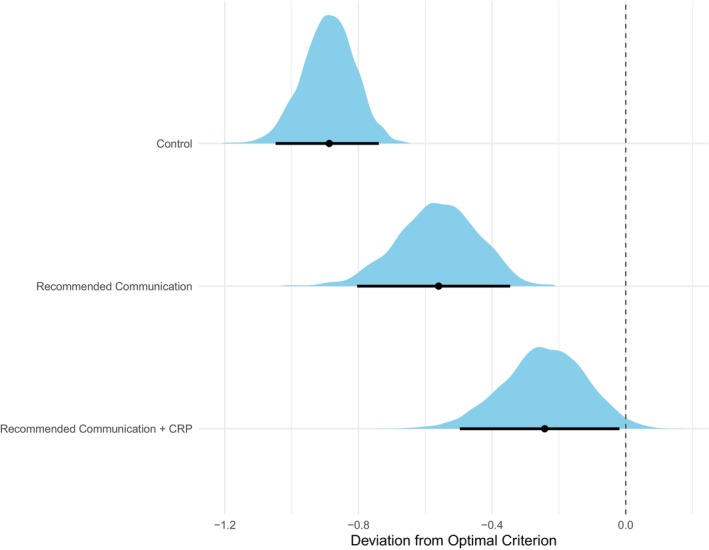
Deviations from the optimal criterion across conditions. The figure shows the mean posterior deviations from the optimal criterion across the three conditions. The dashed grey line represents the optimal criterion location, the black dots represent the mean deviations, while the bold black lines represent the 95% credible intervals.

The distribution of antibiotic responses across conditions is shown in Figure 3 and Table 4. Participants in the control condition displayed the most liberal antibiotic bias (i.e., the lowest threshold for deciding antibiotics were needed), mean posterior criterion = 0.98, 95% CI [0.85, 1.12] and mean deviation from optimal criterion (calculated relative to the optimal decision threshold for a 25% prior probability of ‘antibiotics‐needed’ responses) = −0.89, 95% CI [−1.05, −0.74]. By contrast, participants in the recommended communication condition displayed a less liberal bias (i.e., a higher threshold for deciding antibiotics were needed), mean posterior criterion = 2.32, 95% CI [2.12, 2.52]) and mean deviation from optimal criterion = −0.56, 95% CI [−0.81, −0.34], while those in the recommended communication and CRP condition displayed the highest threshold for deciding antibiotics were needed, mean criterion = 2.71, 95% CI [2.49, 2.95] and mean deviation from optimal criterion = −0.24, 95% CI [−0.49, −0.01]. The posterior means and their associated credible intervals for both bias and sensitivity parameters in the three conditions are shown in Table 3, and the deviations from the optimal criterion are shown in Figure 4.

**TABLE 3:** Summary statistics of the signal detection parameters across conditions.

Metrics	Control	Recommended communication	Recommended communication + CRP
*d*‐prime	3.01 [2.71, 3.36]	5.34 [4.89, 5.85]	5.50 [5.05, 6.00]
Criterion	0.98 [0.85, 1.12]	2.32 [2.13, 2.52]	2.71 [2.48, 2.95]
Optimal criterion	1.87 [1.76, 2.01]	2.88 [2.67, 3.11]	2.95 [2.74, 3.18]
Deviation from optimal	−0.89 [−1.05, −0.74]	−0.56 [−0.81, −0.34]	−0.24 [−0.49, −0.01]

*Note*: The table shows the group‐level signal detection model parameters, bias (criterion, i.e., the distance of the decision criterion from the noise distribution; a higher criterion value denotes a less liberal bias), sensitivity (*d*‐prime, i.e., the distance between the signal and noise distribution; a higher d‐prime value denotes higher sensitivity), the optimal criterion location (calculated for a prior probability of 0.25 ‘antibiotics‐needed’ responses) and the mean deviation from the optimal criterion along with their associated 95% credible intervals across conditions.

**TABLE 4:** Percentage of antibiotic responses by condition and scenario type.

Conditions	Scenario type	% Yes	% No
Control	NN	3.1	96.9
Recommended communication	NN	2.8	97.2
Recommended communication + CRP	NN	1.3	98.7
Control	UN	42.7	57.3
Recommended communication	UN	8.9	91.1
Recommended communication + CRP	UN	5.2	94.8
Control	UY	84.8	15.2
Recommended communication	UY	96.9	3.1
Recommended communication + CRP	UY	95.6	4.4
Control	YY	94.9	5.1
Recommended communication	YY	98.6	1.4
Recommended communication + CRP	YY	96.4	3.6

*Note*: The table shows the proportion of antibiotic responses (no vs yes) in the four scenario types: certain antibiotics are not needed (NN), uncertain antibiotics are not needed (UN), uncertain antibiotics are needed (UY) and certain antibiotics are needed (YY) in the control, recommended communication and recommended communication and CRP conditions.

**FIGURE 5:**
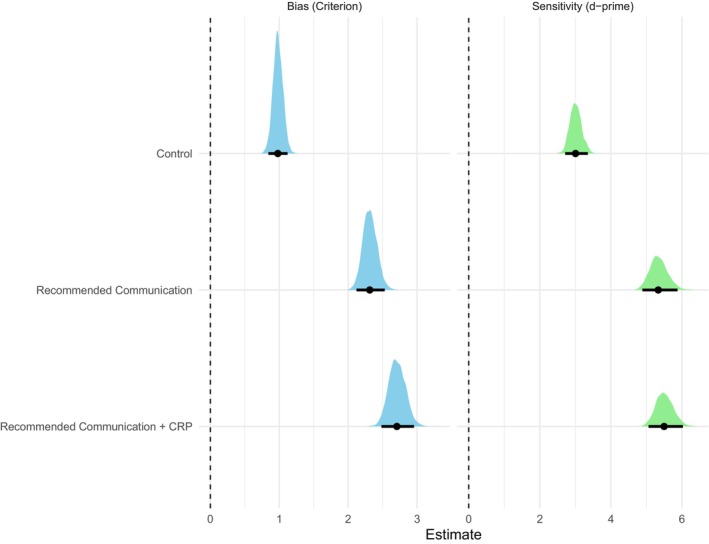
Posterior estimates for bias and sensitivity across conditions. The figure shows the posterior mean estimates for bias (criterion, the distance of the decision criterion from the noise distribution; a higher criterion value denotes a less liberal bias) and sensitivity (d‐prime, the distance between the signal and noise distribution; a higher d‐prime value denotes higher sensitivity) across the three conditions. The black dots represent the mean estimates, while the bold black lines represent the 95% credible intervals.

Aligned with our pre‐registered predictions, participants' antibiotic bias (i.e., their decision criterion) varied significantly across the three conditions in the predicted direction. Specifically, participants in the control condition displayed significantly more liberal bias compared to those in the recommended communication condition, Δ*c* = −1.34, 95% CI [−1.57, −1.11] and the recommended communication and CRP condition, Δ*c* = −1.73, 95% CI [−1.99, −1.48], thus confirming both Hypotheses 1 and 3. Moreover, participants in the recommended communication condition similarly displayed a more liberal antibiotic bias compared to participants in the recommended communication and CRP condition, Δ*c* = −0.39, 95% CI [−0.65, 0.13], therefore confirming Hypothesis 2. The differences in bias and sensitivity across the three conditions, along with their 95% associated credible intervals, are shown in Figure 5.

### Sensitivity

Participants' sensitivity (i.e., their ability to discriminate between the different clinical scenarios whether antibiotics are needed or not) was the lowest in the control condition, mean sensitivity = 3.01, 95% CI [2.71, 3.34]. In contrast, participants' sensitivity was increased in the recommended communication condition, mean sensitivity = 5.34, 95% CI [4.90, 5.85], while it was the highest in the recommended communication and CRP condition, mean sensitivity = 5.50, 95% CI [5.05, 5.99] (see Table 3).

As expected, participants overall displayed significantly lower sensitivity in the control condition compared to both the recommended communication condition, Δ*d*'= 2.34, 95% CI [1.92, 2.79], and the recommended communication and CRP condition, Δ*d*'= 2.49, 95% CI [2.08, 2.95]. Thus, this finding confirms Hypothesis 4, which predicted that participants would display a reduced ability to discriminate correctly between the different scenarios of whether antibiotics are needed or not in the control condition compared to either the recommended communication condition or the recommended communication and CRP condition (see Figure 5).

Overall, all four pre‐registered hypotheses were supported. Participants in the control condition displayed the highest bias towards expecting antibiotics, while providing recommended information about the nature of the illness and the efficacy of antibiotics significantly reduced the antibiotic bias. Moreover, further supplementing the communication intervention with diagnostic CRP point‐of‐care test results yielded the greatest reduction in liberally biased antibiotic expectations. In terms of sensitivity, both communication intervention conditions significantly enhanced diagnostic sensitivity compared to the control, showing that communication strategies, especially when combined with diagnostic test results, are effective in reducing participants' diagnostic uncertainty and therefore their antibiotic expectations.

## DISCUSSION

The present study used a signal detection theory framework to experimentally investigate how different communication interventions can reduce people's antibiotic expectations. Participants were presented with hypothetical consultations for symptoms of respiratory tract infections and were randomly assigned to either a standard information condition (control condition), recommended information about the nature of the illness and the efficacy of antibiotics (recommended communication condition) or recommended information accompanied by the results of a point‐of‐care test (recommended communication and CRP condition). Consistent with our pre‐registered hypotheses, we found that both communication interventions significantly reduced people's antibiotic expectations by lowering their antibiotic bias and improving their diagnostic sensitivity compared to the control.

Participants in the control condition, who received no additional diagnostic information, demonstrated the most liberally biased antibiotic expectations. In contrast, recommended communication (i.e., clinician explanations about the nature of the illness and the efficacy of antibiotics) reduced these expectations substantially, thus confirming Hypothesis 1 that participants would display a more liberal antibiotic bias in the control condition compared to the recommended communication condition. The findings are aligned with past studies showing that communicating clinical information to patients, such as by clarifying that antibiotics are only useful for bacterial infections, significantly decreased people's antibiotic expectations (Sievert et al., 2024; Sirota et al., 2022; Thorpe et al., 2020a, 2020b, 2021). Moreover, the findings also add to the growing body of evidence showing that education‐based patient interventions are effective at reducing inappropriate antibiotic demand (Madle et al., 2004; Roope et al., 2020; Sirota et al., 2022; Thorpe et al., 2020a, 2020b, 2021).

Participants who additionally received information about the results of a point‐of‐care test along with the recommended communication showed significantly less liberally biased antibiotic expectations compared to the control condition, thus confirming Hypothesis 3, which predicted that a more liberal antibiotic bias would be observed in the control condition compared to the recommended communication and CRP condition. Additionally, we found that participants in the recommended communication and CRP condition displayed less liberal antibiotic expectations compared to the recommended communication condition, thus confirming Hypothesis 2, which predicted a more liberal antibiotic bias in the recommended communication compared to the recommended communication and CRP condition. The findings are consistent with past research, which found that including a diagnostic test result significantly decreased people's antibiotic expectations in the United Kingdom (Thorpe et al., 2020a). These findings also build upon existing work showing that CRP testing and CRP training interventions reduced antibiotic prescriptions in clinical practice (Aabenhus et al., 2014; Andreeva & Melbye, 2014; Cals et al., 2009, 2010; Diederichsen et al., 2000; Gonzales et al., 2011; Huang et al., 2013), but extend it by offering novel experimental evidence that *patients* also respond to this information by adjusting their expectations. To the best of our knowledge, this is the first study to demonstrate that presenting patients with point‐of‐care CRP tests results—alongside clinical diagnostic information—reduces their antibiotic expectations.

In terms of sensitivity, both intervention conditions improved participants' ability to discriminate between clinical scenarios where antibiotics were needed compared to the control group, thus confirming Hypothesis 4 that participants in the control condition would have a lower sensitivity compared to either the recommended communication or the recommended communication and CRP condition. The findings show that clinical communication about the illness and the appropriateness of antibiotics, in conjunction with CRP test results, reduces people's antibiotic expectations by improving their diagnostic discernment, suggesting that improving diagnostic clarity would be an effective strategy for lowering inappropriate antibiotic demand. This is especially important given the significant body of evidence showing that diagnostic uncertainty, including poor understanding of symptoms and infection type, as well as antibiotic use, reliably predicts people's inappropriate expectations for antibiotics (Braun & Fowles, 2000; McNulty et al., 2013, 2019; Thorpe et al., 2021; Welschen et al., 2004), and is in line with research calls for more studies on communication intervention methods to help manage uncertainty (Tarrant & Krockow, 2022).

From a theoretical standpoint, the findings provide novel computational evidence for the effects of communicating illness diagnostic and antibiotic information, as well as CRP test results, at reducing people's antibiotic expectations while using a signal detection theory framework (Lynn & Barrett, 2014; Sirota et al., 2022). By disentangling *bias* (i.e., the propensity to expect antibiotics) from *sensitivity* (i.e., the ability to correctly identify when antibiotics are needed), we show how communication interventions influence these two distinct sources of error underlying people's antibiotic expectations (Batailler et al., 2022), and provide computationally testable evidence for the underlying cognitive mechanisms driving antibiotic demand.

The findings also have important implications for clinical practice. They underscore the value of integrating both clinician communication strategies and low‐cost diagnostic tools into routine consultations. This also aligns with national and international antibiotic stewardship goals and calls for developing scalable, cost‐effective and evidence‐based behavioural interventions to mitigate the spread of antibiotic resistance emergence (World Health Organization, 2023). Our findings suggest that even brief additions—such as evidence‐based, tailored illness diagnostic and antibiotic information and CRP point‐of‐care test results—can improve patients' understanding of their illness and when antibiotics are appropriate and reduce inappropriate antibiotic expectations. This would, in turn, reduce unnecessary antibiotic prescribing by mitigating pressure on clinicians (McNulty et al., 2013; Sirota et al., 2017; Welschen et al., 2004), and contribute to broader efforts aimed at curbing the spread of antibiotic resistance.

### Limitations and future directions

Several limitations deserve readers' attention. First, even though we followed the NICE clinical guidelines for developing the antibiotic scenarios and used realistic, and similar symptomatic medical situations to those validated in prior research (i.e., Madle et al., 2004; Sirota et al., 2017; Sirota et al., 2022; Thorpe et al., 2020a, 2020b; Thorpe et al., 2021), the vignettes were nevertheless hypothetical and participants were not making decisions based on currently experiencing an actual illness. Future research should validate the vignettes on patients currently experiencing symptoms of respiratory tract infections and examine whether the interventions will generalize to actual patient–clinician interactions and prescribing behaviours to provide more robust evidence and enhance ecological validity.

Second, even though we employed a sex‐balanced and demographically diverse UK sample, the non‐random sampling approach limits generalizability, while we also did not explore the role of potential mediating and moderating variables that might affect intervention efficacy. For instance, cultural norms (Ventola, 2015), knowledge and health literacy (McNulty et al., 2022), language (Willis & Chandler, 2019) and access to antibiotics and health care (Huttner et al., 2010) are known to shape antibiotic attitudes. Future research should study the role of these variables to guide the development of more tailored communication strategies.

Third, the study focused exclusively on respiratory tract infections, which have been the predominant focus of public health antibiotic campaigns (Huttner et al., 2010; Thoolen et al., 2012). However, conditions such as ear infections and urinary tract infections are also common sources of inappropriate antibiotic expectations, with recent data showing that many individuals wrongly believe antibiotics are effective for most ear infections, while a significant minority underappreciate their utility for urinary tract infections (McNulty et al., 2022). Future research should therefore test the robustness and generalizability of our findings to other types of infections.

## AUTHOR CONTRIBUTIONS

**Andriana Theodoropoulou:** Conceptualization; investigation; funding acquisition; writing – original draft; methodology; validation; visualization; formal analysis; project administration; data curation; resources; writing – review and editing. **Matteo Lisi:** Methodology; validation; visualization; writing – review and editing; software; formal analysis; data curation; resources; supervision. **Jonathan Rolision:** Writing – review and editing; methodology; validation; resources; supervision. **Miroslav Sirota:** Conceptualization; methodology; validation; writing – review and editing; resources; supervision; project administration.

## CONFLICT OF INTEREST STATEMENT

The authors declare that there are no potential conflicts of interest with respect to the research, authorship and/or publication of this article.

## Supporting information


Data S1.

Table S1.

## Data Availability

The registered protocol, raw data, materials and analysis code are publicly available on OSF at: https://osf.io/fd2y4/?view_only=f5b887fc5d5e4272b465f5c1b1a4e883.

## References

[bjhp70020-bib-0001] Aabenhus, R. , Jensen, J. U. , Jørgensen, K. J. , Hróbjartsson, A. , & Bjerrum, L. (2014). Biomarkers as point‐of‐care tests to guide prescription of antibiotics in patients with acute respiratory infections in primary care. Cochrane Database of Systematic Reviews, (11), CD010130. 10.1002/14651858.CD010130.pub2 25374293

[bjhp70020-bib-0002] Albert, R. H. (2010). Diagnosis and treatment of acute bronchitis. American Family Physician, 82(11), 1345–1350. 21121518

[bjhp70020-bib-0003] Andreeva, E. , & Melbye, H. (2014). Usefulness of C‐reactive protein testing in acute cough/respiratory tract infection: An open cluster‐randomized clinical trial with C‐reactive protein testing in the intervention group. BMC Family Practice, 15(1), 80. 10.1186/1471-2296-15-80 24886066 PMC4016668

[bjhp70020-bib-0004] Arnold, S. R. , & Straus, S. E. (2005). Interventions to improve antibiotic prescribing practices in ambulatory care. Cochrane Database of Systematic Reviews, (4). 10.1002/14651858.CD003539.pub2 PMC7003679 16235325

[bjhp70020-bib-0005] Batailler, C. , Brannon, S. M. , Teas, P. E. , & Gawronski, B. (2022). A signal detection approach to understanding the identification of fake news. Perspectives on Psychological Science, 17(1), 78–98. 10.1177/1745691620986135 34264150

[bjhp70020-bib-0006] Braun, B. L. , & Fowles, J. B. (2000). Characteristics and experiences of parents and adults who want antibiotics for cold symptoms. Archives of Family Medicine, 9(7), 589–595. 10910304 10.1001/archfami.9.7.589

[bjhp70020-bib-0007] Cals, J. W. , Butler, C. C. , Hopstaken, R. M. , Hood, K. , & Dinant, G. J. (2009). Effect of point of care testing for C reactive protein and training in communication skills on antibiotic use in lower respiratory tract infections: Cluster randomised trial. BMJ, 338, b1374. 10.1136/bmj.b1374 19416992 PMC2677640

[bjhp70020-bib-0008] Cals, J. W. L. , Schot, M. J. C. , de Jong, S. A. M. , Dinant, G. J. , & Hopstaken, R. M. (2010). Point‐of‐care C‐reactive protein testing and antibiotic prescribing for respiratory tract infections: A randomized controlled trial. The Annals of Family Medicine, 8(2), 124–133. 10.1370/afm.1090 20212299 PMC2834719

[bjhp70020-bib-0009] Centers for Disease Control and Prevention (U.S.) . (2019). Antibiotic resistance threats in the United States, 2019 [Internet]. Centers for Disease Control and Prevention (U.S.). [cited 2023 Apr 28]. https://stacks.cdc.gov/view/cdc/82532

[bjhp70020-bib-0010] Creer, D. D. , Dilworth, J. P. , Gillespie, S. H. , Johnston, A. R. , Johnston, S. L. , Ling, C. , Patel, S. , Sanderson, G. , Wallace, P. G. , & McHugh, T. D. (2006). Aetiological role of viral and bacterial infections in acute adult lower respiratory tract infection (LRTI) in primary care. Thorax, 61(1), 75–79. 10.1136/thx.2004.027441 16227331 PMC2080713

[bjhp70020-bib-0011] Davies, S. C. (2018). Reducing inappropriate prescribing of antibiotics in English primary care: Evidence and outlook. Journal of Antimicrobial Chemotherapy, 73(4), 833–834. 10.1093/jac/dkx535 29490040

[bjhp70020-bib-0012] Diederichsen, H. Z. , Skamling, M. , Diederichsen, A. , Grinsted, P. , Antonsen, S. , Petersen, P. H. , Munck, A. P. , & Kragstrup, J. (2000). Randomised controlled trial of CRP rapid test as a guide to treatment of respiratory infections in general practice. Scandinavian Journal of Primary Health Care, 18(1), 39–43. 10.1080/02813430050202541 10811042

[bjhp70020-bib-0013] Gelman, A. , & Rubin, D. B. (1992). Inference from iterative simulation using multiple sequences. Statistical Science, 7(4), 457–472. 10.1214/ss/1177011136

[bjhp70020-bib-0014] Gonzales, R. , Aagaard, E. M. , Camargo, C. A., Jr. , Ma, O. J. , Plautz, M. , Maselli, J. H. , McCulloch, C. E. , Levin, S. K. , & Metlay, J. P. (2011). C‐reactive protein testing does not decrease antibiotic use for acute cough illness when compared to a clinical algorithm. The Journal of Emergency Medicine, 41(1), 1–7. 10.1016/j.jemermed.2008.06.021 19095403

[bjhp70020-bib-0015] Gonzales, R. , Bartlett, J. G. , Besser, R. E. , Cooper, R. J. , Hickner, J. M. , Hoffman, J. R. , & Sande, M. A. (2001). Principles of appropriate antibiotic use for treatment of acute respiratory tract infections in adults: Background, specific aims, and methods. Annals of Internal Medicine, 134(6), 479–486. 10.7326/0003-4819-134-6-200103200-00013 11255524

[bjhp70020-bib-0016] Goossens, H. , Ferech, M. , Vander Stichele, R. , & Elseviers, M. (2005). Outpatient antibiotic use in Europe and association with resistance: A cross‐national database study. The Lancet, 365(9459), 579–587. 10.1016/S0140-6736(05)17907-0 15708101

[bjhp70020-bib-0017] Gulliford, M. C. , Dregan, A. , Moore, M. V. , Ashworth, M. , van Staa, T. , McCann, G. , Charlton, J. , Yardley, L. , Little, P. , & McDermott, L. (2014). Continued high rates of antibiotic prescribing to adults with respiratory tract infection: Survey of 568 UK general practices. BMJ Open, 4(10), e006245. 10.1136/bmjopen-2014-006245 PMC4212213 25348424

[bjhp70020-bib-0018] Huang, Y. , Chen, R. , Wu, T. , Wei, X. , & Guo, A. (2013). Association between point‐of‐care CRP testing and antibiotic prescribing in respiratory tract infections: A systematic review and meta‐analysis of primary care studies. British Journal of General Practice, 63(616), e787–e794. 10.3399/bjgp13X674477 PMC3809432 24267862

[bjhp70020-bib-0019] Huttner, B. , Goossens, H. , Verheij, T. , & Harbarth, S. (2010). Characteristics and outcomes of public campaigns aimed at improving the use of antibiotics in outpatients in high‐income countries. The Lancet Infectious Diseases, 10(1), 17–31. 20129146 10.1016/S1473-3099(09)70305-6

[bjhp70020-bib-0020] Jain, S. , Self, W. H. , Wunderink, R. G. , Fakhran, S. , Balk, R. , Bramley, A. M. , Reed, C. , Grijalva, C. G. , Anderson, E. J. , Courtney, D. M. , & Chappell, J. D. (2015). Community‐acquired pneumonia requiring hospitalization among US adults. New England Journal of Medicine, 373(5), 415–427. 26172429 10.1056/NEJMoa1500245 PMC4728150

[bjhp70020-bib-0021] John, A. S. , Hopstaken, R. , Tirimacco, R. , Audehm, R. , & Price, C. P. (2022). Implementing point‐of‐care CRP testing for better diagnosis of acute respiratory infections. British Journal of General Practice, 72(715), 87–88. 10.3399/bjgp22X718517 PMC8813101 35091415

[bjhp70020-bib-0022] Kaman, W. E. , Andrinopoulou, E.‐R. , & Hays, J. P. (2013). Perceptions of point‐of‐care infectious disease testing among European medical personnel, point‐of‐care test kit manufacturers, and the general public. Patient Preference and Adherence, 7, 559–577. 10.2147/PPA.S44889 23814465 PMC3693915

[bjhp70020-bib-0023] Knoblauch, K. , & Maloney, L. T. (2012). Modeling psychophysical data in R. Springer Science & Business Media.

[bjhp70020-bib-0024] Köchling, A. , Löffler, C. , Reinsch, S. , Hornung, A. , Böhmer, F. , Altiner, A. , & Chenot, J.‐F. (2018). Reduction of antibiotic prescriptions for acute respiratory tract infections in primary care: A systematic review. Implementation Science, 13(1), 1–25. 10.1186/s13012-018-0732-y 29554972 PMC5859410

[bjhp70020-bib-0025] Lim, W. S. , Baudouin, S. V. , George, R. C. , Hill, A. T. , Jamieson, C. , Le Jeune, I. , Macfarlane, J. T. , Read, R. C. , Roberts, H. J. , Levy, M. L. , Wani, M. , Woodhead, M. A. , & Pneumonia Guidelines Committee of the BTS Standards of Care Committee . (2009). BTS guidelines for the management of community‐acquired pneumonia in adults: Update 2009. Thorax, 64(Suppl 3), iii1–iii55. 10.1136/thx.2009.121434 19783532

[bjhp70020-bib-0026] Little, P. , Stuart, B. , Francis, N. , Douglas, E. , Tonkin‐Crine, S. , Anthierens, S. , Cals, J. W. , Melbye, H. , Santer, M. , Moore, M. , Coenen, S. , Butler, C. , Hood, K. , Kelly, M. , Godycki‐Cwirko, M. , Mierzecki, A. , Torres, A. , Llor, C. , Davies, M. , … GRACE consortium . (2013). Effects of internet‐based training on antibiotic prescribing rates for acute respiratory‐tract infections: A multinational, cluster, randomised, factorial, controlled trial. The Lancet, 382(9899), 1175–1182. 10.1016/S0140-6736(13)60994-0 PMC3807804 23915885

[bjhp70020-bib-0027] Little, P. , Stuart, B. , Francis, N. , Douglas, E. , Tonkin‐Crine, S. , Anthierens, S. , Cals, J. W. L. , Melbye, H. , Santer, M. , Moore, M. , Coenen, S. , Butler, C. C. , Hood, K. , Kelson, M. , Godycki‐Cwirko, M. , Mierzecki, A. , Torres, A. , Llor, C. , Davies, M. , … GRACE consortium . (2019). Antibiotic prescribing for acute respiratory tract infections 12 months after communication and CRP training: A randomized trial. The Annals of Family Medicine, 17(2), 125–132. 10.1370/afm.2356 30858255 PMC6411389

[bjhp70020-bib-0028] Lynn, S. K. , & Barrett, L. F. (2014). “Utilizing” signal detection theory. Psychological Science, 25(9), 1663–1673. 10.1177/0956797614541991 25097061 PMC4304641

[bjhp70020-bib-0029] Macfarlane, J. , Holmes, W. , Gard, P. , Thornhill, D. , Macfarlane, R. , & Hubbard, R. (2002). Reducing antibiotic use for acute bronchitis in primary care: Blinded, randomised controlled trial of patient information leaflet. BMJ, 324(7329), 91. 10.1136/bmj.324.7329.91 11786454 PMC64506

[bjhp70020-bib-0030] Madle, G. , Kostkova, P. , Mani‐Saada, J. , Weinberg, J. , & Williams, P. (2004). Changing public attitudes to antibiotic prescribing: Can the internet help? Journal of Innovation in Health Informatics, 12(1), 19–26. 10.14236/jhi.v12i1.104 15140349

[bjhp70020-bib-0031] McNulty, C. , Read, B. , Quigley, A. , Verlander, N. Q. , & Lecky, D. M. (2022). What the public in England know about antibiotic use and resistance in 2020: A face‐to‐face questionnaire survey. BMJ Open, 12(4), e055464. 10.1136/bmjopen-2021-055464 PMC8987214 35387816

[bjhp70020-bib-0032] McNulty, C. A. , Nichols, T. , French, D. P. , Joshi, P. , & Butler, C. C. (2013). Expectations for consultations and antibiotics for respiratory tract infection in primary care: The RTI clinical iceberg. British Journal of General Practice, 63(612), e429–e436. 10.3399/bjgp13X669149 PMC3693799 23834879

[bjhp70020-bib-0033] McNulty, C. A. M. , Collin, S. M. , Cooper, E. , Lecky, D. M. , & Butler, C. C. (2019). Public understanding and use of antibiotics in England: Findings from a household survey in 2017. BMJ Open, 9(10), e030845. 10.1136/bmjopen-2019-030845 PMC6830627 31662380

[bjhp70020-bib-0034] NICE . (2019a). NICE. Overview|cough (acute): Antimicrobial prescribing|guidance|NICE [Internet]. NICE. https://www.nice.org.uk/guidance/ng120

[bjhp70020-bib-0035] NICE . (2019b). NICE. Overview|pneumonia (community‐acquired): Antimicrobial prescribing|guidance|NICE [Internet]. NICE. https://www.nice.org.uk/guidance/ng138

[bjhp70020-bib-0036] NICE . (2021). Clinical knowledge summaries (CKS) | chest infections—adult: Management NICE [Internet]. NICE. https://cks.nice.org.uk/topics/chest‐infections‐adult/management/

[bjhp70020-bib-0037] O'Neill, J. (2014). Antimicrobial resistance: Tackling a crisis for the health and wealth of nations. Review of Antimicrobial Resistance [Internet] https://amr‐review.org/

[bjhp70020-bib-0038] O'Neill, J. (2016). Tackling drug‐resistant infections globally: Final report and recommendations [Internet]. Apo. https://apo.org.au/node/63983

[bjhp70020-bib-0039] Roope, L. S. , Tonkin‐Crine, S. , Herd, N. , Michie, S. , Pouwels, K. B. , Castro‐Sanchez, E. , Sallis, A. , Hopkins, S. , Robotham, J. V. , Crook, D. W. , & Peto, T. (2020). Reducing expectations for antibiotics in primary care: A randomised experiment to test the response to fear‐based messages about antimicrobial resistance. BMC Medicine, 18(1), 110. 10.1186/s12916-020-01553-6 32321478 PMC7178623

[bjhp70020-bib-0040] Shapiro, D. J. , Hicks, L. A. , Pavia, A. T. , & Hersh, A. L. (2014). Antibiotic prescribing for adults in ambulatory care in the USA, 2007–09. Journal of Antimicrobial Chemotherapy, 69(1), 234–240. 10.1093/jac/dkt301 23887867

[bjhp70020-bib-0041] Sievert, E. D. , Korn, L. , Gross, M. , Santana, A. P. , Böhm, R. , & Betsch, C. (2024). Communicating diagnostic uncertainty reduces expectations of receiving antibiotics: Two online experiments with hypothetical patients. Applied Psychology. Health and Well‐Being, 16(3), 1459–1478. 10.1111/aphw.12536 38500005

[bjhp70020-bib-0042] Sirota, M. , Round, T. , Samaranayaka, S. , & Kostopoulou, O. (2017). Expectations for antibiotics increase their prescribing: Causal evidence about localized impact. Health Psychology, 36(4), 402–409. https://psycnet.apa.org/doi/10.1037/hea0000456 28206788 10.1037/hea0000456

[bjhp70020-bib-0043] Sirota, M. , Thorpe, A. , & Juanchich, M. (2022). Explaining and reducing the public's expectations of antibiotics: A utility‐based signal detection theory approach. Journal of Applied Research in Memory and Cognition, 11, 587–597. https://psycnet.apa.org/doi/10.1037/mac0000027

[bjhp70020-bib-0044] Tarrant, C. , & Krockow, E. M. (2022). Antibiotic overuse: Managing uncertainty and mitigating against overtreatment. BMJ Quality and Safety, 31(3), 163–167. 10.1136/bmjqs-2021-013615 34285113

[bjhp70020-bib-0045] Thoolen, B. , de Ridder, D. , & van Lensvelt‐Mulders, G. (2012). Patient‐oriented interventions to improve antibiotic prescribing practices in respiratory tract infections: A meta‐analysis. Health Psychology Review, 6(1), 92–112. 10.1080/17437199.2011.552061

[bjhp70020-bib-0046] Thorpe, A. , Sirota, M. , Juanchich, M. , & Orbell, S. (2020a). ‘Always take your doctor's advice’: Does trust moderate the effect of information on inappropriate antibiotic prescribing expectations? British Journal of Health Psychology, 25(2), 358–376. 10.1111/bjhp.12411 32196870

[bjhp70020-bib-0047] Thorpe, A. , Sirota, M. , Juanchich, M. , & Orbell, S. (2020b). Action bias in the public's clinically inappropriate expectations for antibiotics. Journal of Experimental Psychology: Applied, 26(3), 422–431. 32271052 10.1037/xap0000269

[bjhp70020-bib-0048] Thorpe, A. , Sirota, M. , Orbell, S. , & Juanchich, M. (2021). Effect of information on reducing inappropriate expectations and requests for antibiotics. British Journal of Psychology, 112(3), 804–827. 10.1111/bjop.12494 33543779

[bjhp70020-bib-0049] Tonkin‐Crine, S. , Anthierens, S. , Francis, N. A. , Brugman, C. , Fernandez‐Vandellos, P. , Krawczyk, J. , Llor, C. , Yardley, L. , Coenen, S. , Godycki‐Cwirko, M. , Butler, C. C. , Verheij, T. J. M. , Goossens, H. , Little, P. , Cals, J. W. , & GRACE INTRO team . (2014). Exploring patients' views of primary care consultations with contrasting interventions for acute cough: A six‐country European qualitative study. NPJ Primary Care Respiratory Medicine, 24(1), 1–6. 10.1038/npjpcrm.2014.26 PMC4373386 25030621

[bjhp70020-bib-0050] Tonkin‐Crine, S. K. , San Tan, P. , van Hecke, O. , Wang, K. , Roberts, N. W. , McCullough, A. , Hansen, M. P. , Butler, C. C. , & Del Mar, C. B. (2017). Clinician‐targeted interventions to influence antibiotic prescribing behaviour for acute respiratory infections in primary care: An overview of systematic reviews. Cochrane Database of Systematic Reviews, 9, CD012252. 10.1002/14651858.CD012252.pub2 28881002 PMC6483738

[bjhp70020-bib-0051] Ventola, C. L. (2015). The antibiotic resistance crisis: Part 1: Causes and threats. Pharmacy and Therapeutics, 40(4), 277–283. 25859123 PMC4378521

[bjhp70020-bib-0052] Welschen, I. , Kuyvenhoven, M. , Hoes, A. , & Verheij, T. (2004). Antibiotics for acute respiratory tract symptoms: Patients' expectations, GPs' management and patient satisfaction. Family Practice, 21(3), 234–237. 10.1093/fampra/cmh303 15128681

[bjhp70020-bib-0053] Willis, L. D. , & Chandler, C. (2019). Quick fix for care, productivity, hygiene and inequality: Reframing the entrenched problem of antibiotic overuse. BMJ Global Health, 4(4), e001590. 10.1136/bmjgh-2019-001590 PMC6703303 31497315

[bjhp70020-bib-0054] World Health Organization . (2014). Antimicrobial resistance: Global report on surveillance [Internet]. World Health Organization. [cited 2023 Apr 28]. https://apps.who.int/iris/handle/10665/112642

[bjhp70020-bib-0055] World Health Organization . (2023). Global research agenda for antimicrobial resistance in human health. WHO. https://www.who.int/publications/m/item/global‐research‐agenda‐for‐antimicrobial‐resistance‐in‐human‐health

[bjhp70020-bib-0056] Worrall, G. (2008). Acute bronchitis. Canadian Family Physician, 54(2), 238–239. 18272643 PMC2278319

