# Supplementary material for: The effects of communicating illness diagnostic and treatment information and C‐reactive protein test results on people's antibiotic expectations
Source: Br J Health Psychol. 2025 Sep 2;30(3):e70020. doi: 10.1111/bjhp.70020 (PMC12403044; doi:10.1111/bjhp.70020)
Supplement: Supplementary file 1 — Data S1. [file BJHP-30-0-s002.docx]

Supplementary Materials

Registered Report Design Planner

| Question | Hypothesis | Sampling plan (e.g. power analysis) | Analysis Plan | Interpretation |
| --- | --- | --- | --- | --- |
| Is there a difference between the control and the recommended communication condition in participants’ antibiotic bias? | We hypothesize that participants will display a more liberal antibiotic expectation bias (i.e., resulting in increased antibiotic expectations relative to the criterion location that maximises accuracy) in the control condition compared to the recommended communication condition (Hypothesis 1) | Based on power simulations for finding an effect size of Cohen’s *d* = 0.2 for the differences in the criterion between the control and the recommended communication condition (and using data from our prior studies to model the distribution of random effects and sample simulated participants) assuming α = .05, a sample size of *N* = 250 gives an estimated statistical power of around 90%. We will therefore aim to recruit a minimum of 250 valid responses per condition. | We will run a multilevel Bayesian generalized linear model (with a probit link function, thus equivalent to a signal detection theory model with a Gaussian-distributed latent decision variable) to estimate the group-level signal detection model parameters: bias (the distance of the decision criterion from the noise distribution) and sensitivity (the distance between signal and noise distribution, expressed in standard deviations, also referred to as d'). We will estimate the signal detection theory parameters for the three information provision conditions (control, recommended communication, recommended communication and CRP) to test whether antibiotic expectations (i.e., the location of the decision criterion) change as a function of the information provided. We will estimate the 95% Bayesian credible intervals of the difference in the criterion between the three information provision conditions. | Communicating information regarding the nature of the illness and the efficacy of antibiotics reduces people’s antibiotic expectations compared to communicating only information regarding their symptoms and physical examination. |
| Is there a difference between the recommended communication condition and the recommended communication and CRP condition in participants’ antibiotic bias? | We expect that participants will display a more liberal antibiotic expectation bias in the recommended communication condition compared to the recommended communication and CRP condition (Hypothesis 2) | Based on power simulations for finding an effect size of Cohen’s *d* = 0.2 for the differences in the criterion between the recommended communication and the recommended communication and CRP condition assuming α = .05, a sample size of *N* = 250 gives an estimated statistical power of around 80%. We will therefore aim to recruit a minimum of 250 valid responses per condition. | We will run a multilevel Bayesian generalized linear model (with a probit link function, thus equivalent to a signal detection theory model with a Gaussian-distributed latent decision variable) to estimate the group-level signal detection model parameters: bias and sensitivity. We will estimate the signal detection theory parameters for the three information provision conditions (control, recommended communication, recommended communication and CRP) to test whether antibiotic expectations (i.e., the location of the decision criterion) change as a function of the information provided. We will estimate the 95% Bayesian credible intervals of the difference in the criterion between the three information provision conditions. | Communication information regarding the nature of the illness and the efficacy of antibiotics, as well as the results of a CRP test, reduces people’s antibiotic expectations further than communicating only information regarding illness aetiology and antibiotics efficacy. |
| Is there a difference between the control condition and the recommended communication and CRP condition in participants’ bias? | We hypothesize that participants will display a more liberal antibiotic expectation bias in the control condition compared to the recommended communication and CRP condition (Hypothesis 3) | Based on power simulations for finding an effect size of Cohen’s *d* = 0.4 for the differences in the criterion between the control and the recommended communication and CRP condition assuming α = .05, a sample size of *N* = 250 gives an estimated statistical power of around 100%. We will therefore aim to recruit a minimum of 250 valid responses per condition. | We will run a multilevel Bayesian generalized linear model (with a probit link function, thus equivalent to a signal detection theory model with a Gaussian-distributed latent decision variable) to estimate the group-level signal detection model parameters: bias and sensitivity. We will estimate the signal detection theory parameters for the three information provision conditions (control, recommended communication, recommended communication and CRP) to test whether antibiotic expectations (i.e., the location of the decision criterion) change as a function of the information provided. We will estimate the 95% Bayesian credible intervals of the difference in the criterion between the three information provision conditions. | Communicating information regarding the nature of the illness and the efficacy of antibiotics, as well as the results of a CRP test, reduces people’s antibiotic expectations compared to communicating only information regarding their symptoms and physical examination. |
| Is there a difference between the control and at least one of the two experimental conditions in participants’ antibiotic sensitivity? | We expect that participants will display lower sensitivity (i.e., reduced ability to discriminate correctly whether antibiotics are needed or not) in the control condition compared to either the recommended communication condition or the recommended communication and CRP condition (Hypothesis 4) | Based on power simulations for finding an effect size of Cohen’s *d* = 0.2 for the differences in the sensitivity between the control condition and the recommended communication or the recommended communication and CRP condition assuming α = .05, a sample size of *N* = 250 gives an estimated statistical power of more than 90%. We will therefore aim to recruit a minimum of 250 valid responses per condition. | We will run a multilevel Bayesian generalized linear model (with a probit link function, thus equivalent to a signal detection theory model with a Gaussian-distributed latent decision variable) to estimate the group-level signal detection model parameters: bias and sensitivity. We will estimate the signal detection theory parameters for the three information provision conditions (control, recommended communication, recommended communication and CRP) to test whether antibiotic expectations (i.e., the location of the decision criterion) change as a function of the information provided. We will estimate the 95% Bayesian credible intervals of the difference in the criterion between the three information provision conditions. | Communicating information regarding the nature of the illness and the efficacy of antibiotics or the results of a CRP test improves people’s ability to discriminate whether antibiotics are needed or not compared to communicating only information regarding their symptoms and physical examination. |
