# Supplementary material for: The effects of communicating illness diagnostic and treatment information and C‐reactive protein test results on people's antibiotic expectations
Source: Br J Health Psychol. 2025 Sep 2;30(3):e70020. doi: 10.1111/bjhp.70020 (PMC12403044; doi:10.1111/bjhp.70020)
Supplement: Supplementary file 2 — Table S1. [file BJHP-30-0-s001.docx]

**Table S1**

*Posterior Estimates Under Alternative Priors*

| **Parameter** | **Original Prior (Median ± MAD_SD)** | **Wider Prior  (Median ± MAD_SD)** | **T Prior  (Median ± MAD_SD)** |
| --- | --- | --- | --- |
| (Intercept) | -1.0 ± 0.1 | -1.0 ± 0.1 | -1.0 ± 0.1 |
| anti_needed | 3.0 ± 0.2 | 3.0 ± 0.2 | 3.0 ± 0.2 |
| condition1 | -1.3 ± 0.1 | -1.4 ± 0.1 | -1.3 ± 0.1 |
| condition2 | -1.7 ± 0.1 | -1.8 ± 0.1 | -1.7 ± 0.1 |
| anti_needed:condition1 | 2.3 ± 0.2 | 2.4 ± 0.2 | 2.3 ± 0.2 |
| anti_needed:condition2 | 2.5 ± 0.2 | 2.6 ± 0.2 | 2.5 ± 0.2 |

*Note.* Table S1 shows the posterior medians and median absolute deviations (MAD_SD) for key fixed effects for each prior specification (original prior = normal priors with a mean of zero and a standard deviation of one; wider prior = priors with same means as the original, but with standard deviations doubled; t prior = priors from a t-distribution with 3 degrees of freedom).
